# Sex-differences in excess death risk during the COVID-19 pandemic: an analysis of the first wave across Italian regions. What have we learned?

**DOI:** 10.1186/s41118-022-00172-8

**Published:** 2022-08-06

**Authors:** Silvia Rizzi, Cosmo Strozza, Virginia Zarulli

**Affiliations:** Interdisciplinary Centre on Population Dynamics, University of Southern Denmark, Odense, Denmark

**Keywords:** Sex-differences in excess death, Viral respiratory infectious diseases, COVID-19, Italian regions, Mortality forecast

## Abstract

In this commentary, we bring together knowledge on sex-differences in excess death during the first wave of the COVID-19 pandemic in Italy, one of the most hit European countries. We zoom into Italian regions to account for the spatial gradient of the spread of the virus. Analyses of excess death by sex during the COVID-19 pandemic have been possible thanks to weekly mortality data released by national statistical offices, mainly in developed countries. The general finding is that males up to 75 years old have been suffering more excess death compared to females. However, the picture is less clear-cut at older ages. During previous epidemics, such as SARS, Swine Flu, and MERS, studies are limited and produce scattered, non-conclusive evidence. Knowledge of the sex-pattern of susceptibility to mortality from virulent respiratory diseases and its interplay with age could improve crisis management during future epidemics and pandemics. National statistical offices should provide weekly mortality data with spatial granularity, disaggregated by sex and age groups, to allow for such analyses.

## Commentary

Studies of mortality during the COVID-19 pandemic found geographical gradients, age gradients, and sex-differences in excess mortality, i.e., mortality above the level expected in normal conditions. Excess death has been widely used as a comprehensive measure to quantify the total impact of the COVID-19 pandemic. The concept of excess mortality captures deaths caused both directly by the virus and indirectly because of the pressure on health care systems and the effects of policy interventions. Excess mortality is more likely to be a valid proxy for a particular cause of death if the shock in the given cause is high enough to push all-cause mortality above its usual range. A shock to a cause must be large in order to associate excess with the cause, and that is why in this paper we focused on the first wave of the COVID-19 pandemic in Italian regions.

The estimation of excess death relies on all-cause mortality data; therefore, it overcomes the limitation of causes of death registrations. Excess death is indeed the difference of the total number of deaths observed and those expected if the health shock had not occurred. Over the course of the COVID-19 pandemic, excess death estimates have been generally stratified by demographic characteristics, such as age, sex, and geographical areas. While the highest excess mortality was generally found at older ages compared to the younger ones, results have been less consistent when it has come to excess mortality by sex. The prevailing view is that, although number of male and female cases are similar, men suffer from higher risk of death compared to women. This sex differential varies markedly by age (Bhopal & Bhopal, 2020). For instance, in Sweden, men suffered greater excess mortality than women in ages up to 75 years, but at older ages, excess mortality was similar for men and women (Modig et al., 2021). In England and Wales, males had larger excess mortality rates compared to females across all age groups, but when excluding the direct COVID-19 deaths, females had higher excess mortality in the 85+ age group (Kontopantelis et al., 2021). In Italy, analyses of excess mortality during the first wave show a situation where, in general, men suffered slightly higher excess mortality than women. Men reached their mortality peak in March 2020, while women suffered more in the following 2 months (Caselli et al., 2021). However, the picture is more nuanced. In the Northern regions of Italy, i.e., those worst hit by the virus, reductions in life expectancy and years of life lost were higher for men than for women (Ghislandi et al., 2020), but excess mortality was sizably higher for men than for women only until age 75, after which excess mortality was similar for both sexes (Gibertoni et al., 2021). At the national level, the pandemic appears to have had only a slightly higher impact in men compared to women (Caselli et al., 2021; Scortichini et al., 2020). The peak of the excess death toll was in March 2020 (Gianicolo et al., 2021), specifically, during the weeks of March 18th for men and March 25th for women (Blangiardo et al., 2020). Furthermore, when distinguishing total excess mortality from excess mortality directly related to COVID-19, only the latter was higher in males than in females, more so in the North than the South, while the total excess mortality was the same for both sexes (Michelozzi et al., 2020). This raises the important question of whether there is a bigger indirect impact on mortality for women than for men. Sudden emergent diseases can often lead to large breakdowns in public health systems, which directly and indirectly affects marginalized and vulnerable sub-populations, including women (Hanson-DeFusco, 2020).

**Fig. 1:**
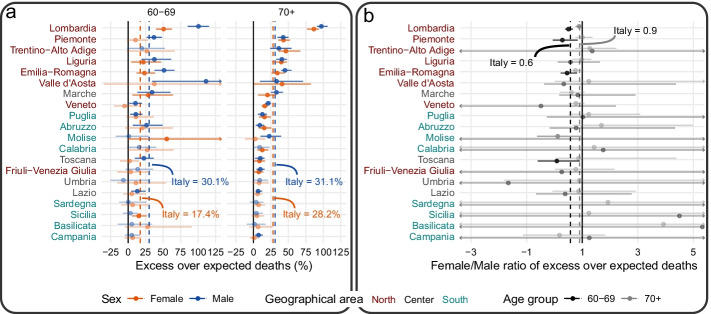
Excess death risk by sex and 20 Italian regions for the age groups 60–69 and 70+ (**a**) and ratio between women’s and men’s excess death risk (**b**). Regions are ordered according to the total value of excess over expected deaths observed for the age group 70+. Geographical area South includes Islands

The not-very-pronounced sex difference in excess mortality during the first wave of COVID-19 in Italy was also confirmed by our application of the method for counterfactual short-term mortality forecasts after mortality shocks (Rizzi & Vaupel, 2021). Using data of monthly all-cause deaths stratified by sex, age group, and Italian region from 2014 through 2020 retrieved from ISTAT (Istat, 2021), we forecasted the expected number of deaths spanning from the beginning of March 2020 through the end of June 2020. The forecast is based on the ratios of the death counts between two parts of an epidemiological year (epiyear). An epiyear starts July 1st and ends June 30th of the following calendar year. Epiyears are used in seasonal mortality analyses because they contain one mortality peak concentrated during the winter months. In our application, each epiyear, analyzed from 2014 through 2020, is divided in two parts: an earlier part that spans from July through February and a later part from March through the end of June, corresponding to period of the first COVID-19 wave. Ratios of deaths in the second part of an epiyear to deaths in the first part of an epiyear are fairly constant over time and proved to be stationary. Therefore, by multiplying the average value of these ratios by the deaths of the first part of epiyear 2019–2020, we forecasted the expected number of deaths if the first COVID-19 wave had not occurred. By subtracting the expected deaths from those observed, we estimated excess deaths. Our estimates’ uncertainty was derived via a bootstrapping procedure to account for the difference between the unknown later/earlier ratio in epiyear 2019–2020 in the absence of COVID-19 and its average value of the previous 5 years used for the counterfactual shortcast. The statistical analysis was performed with R version 4.0.2 (R Core Team, 2020); data and code for full reproducibility can be found on GitHub [will be made available after peer-review]. We present our results (Table 1; Fig. 1, panel a) in terms of excess death risk, i.e., the ratio between excess death estimates and expected deaths, to allow for comparison across regions with different population sizes and structures. At the national level, we found the excess death risk to be 25.0% both for men and women, mostly over age 70. Men generally had more excess deaths compared to women, but the only statistically significant difference between the two sexes occurred for the age categories 60–69 and 70–79 (Table 1). Furthermore, we found major differences at the regional level (age group 60 +), spanning from 100.6% and 97.2% in Lombardia to 5.2% and 7.4% in Campania for men aged 60–69 and 70+, respectively, and from 50.8% and 86.5% in Lombardia to 6.6% and less than 1.4% in Campania for women aged 60–69 and 70+, respectively (Fig. 1, panel a). The figure shows the clear North–South mortality gradient that characterized the first wave of the pandemic in Italy, with Lombardia being the only one where there was a statistically significant difference between the excess mortality of men and women aged 60–69. Furthermore, results are presented as ratio between women’s and men’s excess death risk (Fig. 1, panel b). At the national level, we found a ratio of 0.6 for the age category 60–69. The excess death risk was significantly higher for men in Lombardia, Piemonte, Emilia-Romagna and Toscana. For the age category 70+ the ratio, at the national level, is close to 1 (0.9) and there were no regions with values statistically different from 1. Overall, panel b in Fig. 1 is characterized by high variability, especially for those regions experiencing fewer deaths in the first wave of the pandemic.

**Table 1:** Deaths (observed, expected, excess) and excess death risk by sex and 5 age group for Italy at the national level from March 1st 2020 through 30th June 2020

Sex	Age group	Observed deaths	Expected deaths	Excess deaths	Excess death risk (%)	Prediction interval
Female	0–59	5882	5401	481	8.9	(5.2–12.3%)
	60–69	7764	6602	1162	17.6	(13.7–22.3%)
	70–79	20,058	15,984	4074	25.5	(21.8–30.0%)
	80–89	52,771	40,565	12,206	30.1	(24.0–34.1%)
	90 +	46,773	36,544	10,229	28.0	(20.9–33.6%)
Male	0–59	10,284	9312	972	10.4	(7.2–13.4%)
	60–69	14,580	11,201	3379	30.2	(26.7–33.3%)
	70–79	31,725	23,567	8158	34.6	(32.1–37.1%)
	80–89	49,501	37,632	11,869	31.5	(27.7–35.4%)
	90 +	20,545	16,396	4149	25.3	(21.0–30.3%)

What do we know about sex-differences in excess mortality during other epidemics of viral respiratory infectious diseases?

A study of 27 European populations for the years 2016 through 2020 found that in winter periods, with increased excess mortality driven mainly by influenza, excess death was consistently highest among males, with an overall female/male mortality incidence ratio of 0.7 (Nielsen et al., 2021). The study also found a general linear association between increasing excess mortality and sex-differential excess mortality, most striking for the ages between 15 and 64. Sex differences in hormones, immune responses, behavior, and the broad gendered-based panorama of social determinants of health are likely to play a major role in differences in excess mortality outcomes (Mauvais-Jarvis et al., 2020; Takahashi et al., 2020). In addition, gender differences in excess death could also be driven by the “dry tinder” effect, where the frailer individuals are particularly susceptible to sudden shocks. Higher male excess mortality has been reported in some cases where infectious diseases caused elevated excess mortality (Alghamdi et al., 2014; El Bcheraoui et al., 2018). However, while sex-differences in general mortality have been investigated, there are a limited number of studies that have analyzed male–female difference in excess death during influenza epidemics and the last three major pandemics from respiratory viruses in the pre-COVID era: SARS, Swine Flu, and MERS. The results produced scattered, non-conclusive evidence of little or no sex-differences in excess mortality (Jin et al., 2020; Liu et al., 2017; Rajatonirina et al., 2013; Takahashi & Nagai, 2008). In the case of SARS-Cov, which was discovered in China at the end of 2002 and rapidly spread to Hong Kong and other Asian countries (Chan-Yeung & Xu, 2003), the case-fatality rate was higher among men younger than 75 compared to women (Karlberg et al., 2004). One limitation of excess mortality analysis during epidemics and pandemics is that the health shock must be substantial to link the estimated excess with the cause. When this is not the case, e.g., fatality rate is high but effect on excess mortality is negligible, sex-differences in mortality are better captured by studying the cause of death directly, assuming that misreporting is equal among sexes.

## Conclusions

We have analyzed sex-differences in excess death risk during the first wave of the COVID-19 pandemic in Italy and placed it into the international context. Gender differences in excess risk of death existed, but with a nuanced and non-consistent picture. Even though the results pertain to Italy and cannot be generalized, we brought together the existing knowledge on sex-differences in excess death during the COVID-19 pandemic and other epidemics of viral respiratory infectious diseases, from which, we believe, we can draw important lessons for the future. First, we saw that there is a lack of studies on sex-differences in excess mortality from major respiratory infectious diseases. The WHO Gender Strategy (WHO, 2009) mandates that “the different needs of women and men are considered at all stages of policy and program development”. Despite this mandate, consideration of sex in emerging infectious disease programs is still the exception rather than the rule. A greater understanding of how sex influences the epidemiology of influenza and other respiratory diseases may prove useful for clinical, public health, and government activities that are critical in disease prevention and control. Second, we saw that sex-differences in excess death during the first wave of COVID-19 in Italy were not equally strong throughout the regions and the age spectrum. Similar patterns appeared in other countries. Such subtle and inconsistent differences can be difficult to detect, especially in the first phases of a pandemic. A better knowledge of the sex-pattern of susceptibility to mortality from virulent respiratory diseases and its interplay with age could improve early detection and crisis management during future pandemics. National statistical offices should provide weekly mortality data with spatial granularity, disaggregated by sex and age groups, to allow for such analyses.

## Figures and Tables

**Fig. 2:**
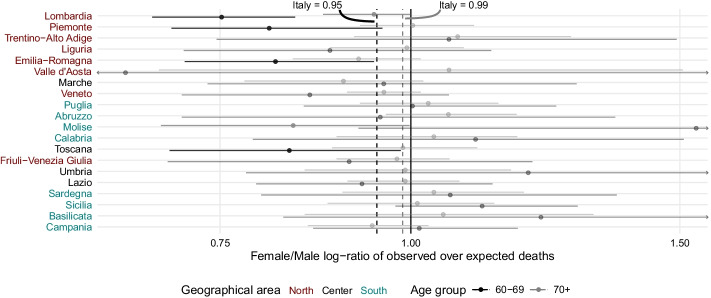
Female/male ratio of observed over expected deaths on log scale by 20 Italian regions for the age groups 60–69 and 70+

## Data Availability

Data are freely available at https://www.istat.it/it/archivio/240401. Data and code for full replicability are available under the authors’ GitHub.

## Appendix

See Fig. 2.
